# Chorea as a Manifestation of Systemic Lupus Erythematosus

**DOI:** 10.7759/cureus.35884

**Published:** 2023-03-07

**Authors:** Teresa Medeiros, Andreia Vilas-Boas, Vanessa Carvalho, Teresa Santos, Ana Pinho

**Affiliations:** 1 Internal Medicine, Hospital Pedro Hispano, Porto, PRT; 2 Internal Medicine, Hospital Luz Arrábida, Vila Nova de Gaia, PRT; 3 Neurology, Hospital Pedro Hispano, Matosinhos, PRT; 4 Nephrology, Hospital Pedro Hispano, Matosinhos, PRT; 5 Internal Medicine, Hospital Pedro Hispano, Matosinhos, PRT

**Keywords:** leukocytoclastic vasculitis (lcv), auto immune disease, sle and lupus nephritis, chorea and hemiballismus, systemic lupus erythromatosus

## Abstract

Systemic lupus erythematosus (SLE) is an autoimmune disease with multisystemic manifestations, including central nervous system involvement. Chorea is a hyperkinetic movement disorder, characterized by involuntary, dance-like and poorly coordinated movements. Acute-onset chorea is a rare neuropsychiatric inaugural manifestation of SLE. This presentation is frequently associated with positive antiphospholipid antibodies, and it usually improves with immunosuppressive treatment.

We report the case of a 20-year-old female, who presented with acute onset left hemichorea and fever. Analysis showed active urine sediment. A detailed anamnesis and evaluation revealed several clinical manifestations suggestive of SLE with multiorgan involvement: neurological, renal, cardiac, hematological, joint and mucocutaneous.

This case emphasizes the importance of keeping a high clinical awareness for rarer presentations of common autoimmune disorders, such as SLE, which can be severe and should be promptly treated. Furthermore, the relevance of SLE in the differential diagnosis of acute-onset movement disorders in young patients is highlighted in this report.

## Introduction

Systemic lupus erythematosus (SLE) is a chronic autoimmune disorder, characterised by the presence of autoantibodies, systemic inflammation and multiorgan involvement, including, less frequently, the central and peripheral nervous system [1-4]. Neuropsychiatric symptoms of SLE (NPSLE) are particularly difficult to identify and diagnose but account for a considerable percentage of manifestations, with an estimated prevalence ranging from 37% to 95%. The lack of uniformity in definitions, nomenclature, and diagnostic criteria contributes to the considerable variability in the estimated prevalence of NPSLE [5-7].

In 1999, the American College of Rheumatology issued a proposal for the nomenclature and definition of NPSLE [4]. The proposal defined 19 syndromes, including movement disorders. Chorea is a hyperkinetic movement disorder characterised by involuntary, dance-like, and unpredictable movements. Chorea, although rare, is well described in SLE, with a cumulative incidence of 0.6% [7]. Other causes may be genetic, vascular, inflammatory, infectious, drug-induced or metabolic. We report a case of acute onset hemichorea as a form of NPSLE.

## Case presentation

A 20-year-old female presented to the emergency department with 24 hours onset of involuntary movements of the left hemibody. She denied being pregnant and had no relevant past medical history or chronic medication, including use of anticonception medication. On admission, she had exuberant choreiform movements of the left hemibody, afflicting the face and limbs, with predominant involvement of the upper limb (Video 1). The remaining neurological examination was unremarkable. Physical examination showed discrete malar erythema, millimetric papular lesions on the right forearm extensor surface, painful oral ulcers, episcleritis of the right eye and signs of arthritis of the right joint. Initial workup revealed pancytopenia, an increased erythrocyte sedimentation rate (ESR), a positive direct Coombs test and leukoerithrocyturia, with normal renal function, as shown in Table 1.

**Video 1:** The patient presented dance-like involuntary movements on the left upper and lower limbs, suggestive of chorea.

**Table 1:** Laboratory results

Haemoglobin	7.0g/dL normocytic normochromic;
White blood cell count	3,160/μL
Platelet count	131K/μL
ESR	71mm/hour
Urea	30mg/dL
Creatinine	0.6mg/dL

Brain computed tomography scan was normal. Haloperidol 0.5mg twice daily was started to manage the disabling movement disorder. A thorough questioning history revealed a vespertine fever in the preceding eight weeks, initially attributed to a cystitis due to the presence of leukoerythrocyturia and treated with antibiotics. Later, due to the emergence of asthenia, weight loss, odynophagia and an inflammatory asymmetric polyarthralgia of the small and large joints, pancytopenia, the case was interpreted as a possible infectious mononucleosis. The patient was monitorised and no specific treatment was initiated.

**Figure 1:**
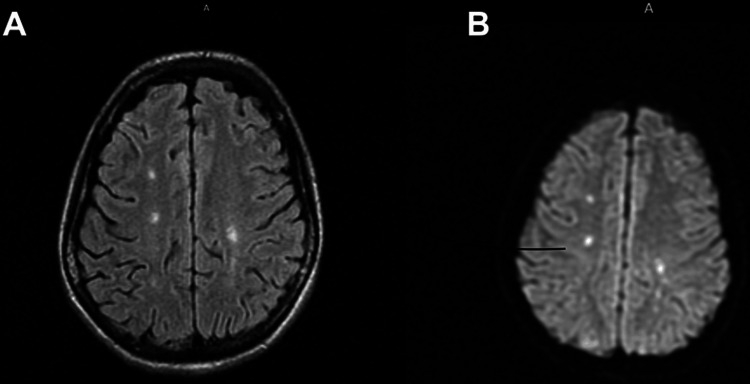
Brain MRI showing white matter hyperintensities on T2-FLAIR (1A) and DWI (1B), compatible with several acute stroke lesions.

The diagnostic approach included an autoimmune workup that showed an anti-nuclear antibody (ANA) titer of 1:1,280 (normal <1: 40), with cytoplasmic staining suggestive of anti-ribosome antibodies; a positive anti-double-stranded DNA and complement consumption with C3 35 mg/dL (normal range [NR] 83-193) and C4 8.4mg/dL (NR 15-57). Antiphospholipid antibodies were negative. Urinalysis showed nephrotic-range proteinuria (4.1g/24h). Chest radiography exhibited an increased cardiothoracic ratio, and a moderate volume pericardial effusion was confirmed by transthoracic echocardiogram. A magnetic resonance imaging (MRI) of the brain showed small T2 hyperintense areas in the subcortical bihemispherical and periventricular white matter (Figures 1A, 1B), compatible with ischemic lesions so aspirin was introduced (100 mg/day). The cytochemical study of the cerebrospinal fluid was unremarkable. Skin biopsy of the forearm lesions demonstrated signs of leukocytoclastic vasculitis and an immunofluorescence pattern consistent with SLE; additionally, a renal biopsy revealed class IV-G (A) lupus nephritis, confirming the SLE diagnosis with a total EULAR/ACR-2019 score of 39 (10 to 51 points) [1].

The patient was started on immunosuppressive treatment - methylprednisolone 1g/day for three days, followed by prednisone 1mg/kg/day and mycophenolate mofetil, up to a dose of 3g/day, with a favourable response. The choreic movements ceased after the first days of hospitalisation, and haloperidol was progressively tapered.

## Discussion

The lack of specificity of imaging abnormalities and the absence of specific biomarkers make the diagnosis of NPSLE difficult, relying essentially on clinical judgement. No complete comprehension of the pathogenesis and the consequent pathophysiological changes to explain the CNS involvement in SLE; yet it is likely that multiple mechanisms coexist immune, inflammatory, and thrombotic/ischemic [8].

Chorea has a wide list of differential diagnoses. In this patient, the acute-onset chorea could be due to vascular, infectious, endocrine/metabolic, toxic or autoimmune causes, even though the asymmetric onset would could normally suggest a structural lesion [8,9]. Nonetheless, a careful anamnesis and adequate interpretation of clinical data and blood analysis soon unveiled a disease with multiple organ involvement, which was most likely of autoimmune origin, considering the patient’s age and clinical presentation.

Chorea affects mainly women (in almost 90% of cases) and may precede the onset of other findings of SLE [10]. The presence of antiphospholipid antibodies is more frequent in patients that develop chorea when compared with other SLE patients (92% vs 25%, respectively) [11]. Most patients have antiphospholipidious antibodies which may be associated with hormonal variations, with increased levels of oestrogen such as pregnancy or the taking of contraceptives (which is not the case), independent of the presence of a vascular event. The pathophysiology of the chorea in lupus is still not well understood. Manifestations can be unilateral even in the absence of structural injury in imaging exams [12].

Concerning imaging studies, MRI is the most common exam undertaken in the investigation of these patients, since it allows the differential diagnosis with other causes - however, it is nonspecific for SLE diagnosis. Although chorea/hemibalism is classically associated with subthalamic nucleo lesions, presentations with hemichora/hemibalism are described in international records in patients with stoke in white substance, provided they affect the nucleo-linking pathways [13]. There are neither SLE-specific changes nor correlations between imaging findings and clinical manifestations and the MRI are normal in 40% of all NPSLE [14]. So, hemichora/chorea is a classic manifestation of neurolupus, lesions outside the ganglios of the base - already described, maybe by lesion of the pathways between the ganglios of the base.

There is no consensus about therapeutic strategies in NPSLE, but the recommendation is a therapy based on the primary pathogenic mechanism: either immunosuppressive treatment or anticoagulation/antiplatelet treatment, depending on the most likely involved pathophysiologic mechanisms. The combination of both treatment options is preferred when both mechanisms are considered a possibility [7]. In the case of persistent chorea, patients may be symptomatically treated with haloperidol or other neuroleptics [3].

## Conclusions

The causal relationship between neuropsychiatric symptoms and SLE is still a challenge and requires the exclusion of other potential causes. In this case, a urinary tract infection is by far more likely in a young and healthy woman than a systemic disease, so the diagnosis was significantly delayed in this patient. Neurological manifestations, although a sign of severe disease, are usually manageable and respond well to immunosuppressive therapy, as observed in our patient.

Our case highlights the crucial importance of a well-conducted interview and clinical observation, and the richness of a thorough differential diagnosis, underlining the importance of maintaining a high index of suspicion. This case illustrates the relevance of SLE in the differential diagnosis of acute-onset movement disorders in young patients. The engagement of a multidisciplinary team is crucial for the successful management of these patients.
